# Therapeutic alliance and dropout in patients with borderline pathology receiving residential dialectical behavior therapy

**DOI:** 10.1186/s12888-023-05061-8

**Published:** 2023-08-18

**Authors:** Carolin Steuwe, Michaela Berg, Martin Driessen, Thomas Beblo

**Affiliations:** Department of Psychiatry and Psychotherapy, Bielefeld University, Bielefeld, Germany

**Keywords:** Borderline Personality Disorder, Dialectical Behavior Therapy, Dropout, Therapeutic alliance

## Abstract

**Background:**

This study focused on the impact of therapeutic alliance on therapy dropout in a naturalistic sample of patients with borderline pathology receiving dialectical behavior therapy (DBT) in a residential setting. We assumed that low therapeutic alliance shortly after admission would be associated with elevated dropout.

**Methods:**

44 participants with borderline pathology (≥ 3 DSM-5 borderline personality disorder criteria) in a residential DBT program completed a quality assurance questionnaire set assessing demographic information, pretreatment psychopathology and therapeutic alliance during the first seven days of their residential stay. Predictors of dropout were investigated using binary logistic regression analyses.

**Results:**

The dropout rate was 34.1% (*n* = 15). In binary logistic regression analyses with variables covering demographic and clinical characteristics, comorbidities and childhood trauma history, only the therapeutic alliance significantly predicted dropout (*z* = -2.371, *p* = .018).

**Conclusions:**

This study supports the importance of therapy process variables, here the therapeutic alliance at the beginning of treatment, as predictors of therapy dropout in borderline pathology. If this finding is replicated, it shows the potential importance of monitoring the therapeutic relationship throughout the therapeutic process. ClinicalTrials.gov Identifier: NCT05289583, retrospectively registered on March 11, 2022.

## Introduction

Although dropout rates in the treatment of Borderline Personality Disorder (BPD) patients are lower than originally expected, dropout remains a substantial problem in psychotherapy [1]. Approximately one third of all BPD patients receiving psychotherapy do not complete the treatment, and there are inconsistent findings regarding the predictors of therapy dropout [2]. In a meta-analysis by Barnicot et al. [1], socio-demographics were consistently non-predictive, while commitment to change, the therapeutic relationship and impulsivity were found to predict dropout. Recent studies have expanded the list of potential predictors in BPD. Gamache et al. [3] found four variables (narcissism, secondary gains, low distress, and cluster A features) to predict dropout in BPD patients. These may be useful to discriminate between different subgroups of patients who drop out prematurely from psychotherapy. In an early study by Gunderson et al. [4] on BPD patients, patients that dropped out were healthier on some baseline measures than those who completed treatment. The type of psychotherapy could influence the dropout rate as well. For example, Waldinger and Gunderson [5] found that patients receiving psychoanalysis were less likely to terminate treatment precipitously than patients receiving intense psychotherapy.

Dialectical Behavioral Therapy [6] is a specialized treatment for BPD and has been shown to be effective in a wide range of studies. It has also proven to be more effective than active control conditions [7]. However, even for DBT, a mean dropout rate of around 28% is found in meta-analyses [7, 8]. Unfortunately, the superiority of DBT is not evident with regard to dropout rates [8]. Research into the factors of dropout is therefore important to successfully deliver this highly effective therapeutic procedure to as many patients as possible.

Similar to studies on dropout in BPD in general, previous research on predictors of dropout from DBT has focused on socio-demographic and clinical variables. Younger age [9, 10], receiving disability benefits [11] and low education [12, 13] were found predictive of dropout in some studies, whereas others could not demonstrate any influence of socio-demographic factors on dropout in DBT [e.g., 14]. Comorbidities, particularly eating disorders and substance use disorders, are frequently found to predict dropout [9, 14–16]. In addition, Herzog et al. [13] found that being diagnosed with an endocrine, metabolic, or nutritional disease or a comorbid recurrent depressive disorder were the predictors of treatment completion. Beyond that, specific symptom domains have significant associations with treatment discontinuation, even if the direction of the effect is not always clear. Fewer lifetime suicide attempts were a protective factor in some studies [17], whereas it increased the risk of dropout in others [15, 16]. Moreover, anxiety [17, 18], bodily pain [13], anger [16, 17], and impulsivity [14] have been shown to predict dropout. Furthermore, non-acceptance of emotional responses [10] and experiential avoidance [17, 19] may be associated with an increased risk for dropout. There are mixed results with regard to childhood trauma. Euler et al. [20] found that emotional abuse during childhood predicted dropout from DBT, whereas the dropout rate was lower in patients who reported childhood emotional neglect. However, Carmona et al. [14] did not find a relation between childhood trauma history and dropout. In our own study [21], similar to Euler et al. [20], a high degree of childhood emotional abuse was associated with premature termination of treatment, whereas physical neglect during childhood was associated with a protective effect on treatment dropout in a residential setting [21]. The latter result was interpreted in the way that basic needs (e.g., food, medical care) which might have been deprived during childhood were fulfilled during the residential treatment, and thereby the likelihood of a preliminary dropout decreased.

Because interpersonal problems represent core difficulties in patients with BPD [22], the therapeutic alliance may be particularly vulnerable and at the same time particularly important in psychotherapeutic work in this group of patients. There is no consensual definition of the term therapeutic alliance; therefore, alliance measures mostly define what is meant by therapeutic alliance [23]. A widely used instrument to capture the therapeutic alliance is the Working Alliance Inventory [24]. It is based on the three constituent components (bonds, goals, and tasks) conceptualized by Bordin [25], which in combination define quality and strength of the therapeutic alliance.

In a meta-analysis identifying the effective factors of dialectical behavior therapy, therapeutic alliance is one of three core mechanisms of change in symptom severity [26]. On the therapeutic relationship, little data is available with regard to dropout in DBT. Chalker et al. [19] found that more frequent phone contacts were associated with a decrease in dropout and an increase in client and therapist satisfaction, thus indicating the relevance of the therapeutic relationship. In a previous study, we found a change of therapists between DBT-briefing and treatment to be associated with an elevated risk of dropout [21]. However, in our study, the quality of the therapeutic relationship was not measured directly. To our knowledge, so far only a study by Wunk et al. [16] has investigated the association between DBT and therapeutic alliance. Here, the last recorded value on the therapeutic relationship before therapy discontinuation was significantly associated with treatment discontinuation (a weak therapeutic alliance predicted dropout).

The potential influence of the therapeutic relationship on dropout rates is also supported by studies investigating the association of therapeutic alliance in psychotherapy across disorders, and in BPD specifically. In a non-diagnosis-specific meta-analysis examining therapeutic alliance in adult individual psychotherapy, a moderately strong relationship can be found between psychotherapy dropout and therapeutic alliance (*d* = 0.55), indicating that a weaker therapeutic relationship leads to an increased risk of dropout [27]. In BPD, an early study by Yeomans et al. [28] supported the assumption that the therapist’s technique, such as therapists’ contribution to a treatment contract and the therapeutic alliance (in psychodynamic psychotherapy), plays a role in preventing dropout. Consistently, Spinhoven et al. [29] showed that negative ratings of therapists and patients, especially in early treatment, were predictive of dropout from schema-focused therapy and transference-focused psychotherapy for borderline personality disorder. The first phase of treatment could therefore be a critical phase for treatment retention. An overview of predictors of dropout across psychological therapies in BPD, extracted from meta-analyses, as well as predictors of dropout in DBT can be found in Table 1.

**Table 1:** Predictors of dropout in BPD across psychotherapy treatments (drawn from meta-analyses only) and specific to DBT

Predictors of dropout	Across psychotherapeutic methods for BPD	In DBT for BPD
Demographics	No impact of age, gender, marital status, living alone, education level, employment status, race, and religion [1]	Younger Age [9, 10] Disability benefits [11] Low education [12, 13] No impact of demographics [14]
Comorbidities	Schizoid personality disorder predictive in one study [1] Greater number of personality disorders [1] No impact in terms of comorbid axis 1 or axis 2 disorders [1]	Eating disorders [9, 14] Substance use disorders [9, 14] Antisocial personality disorder [15] Recurrent depressive disorder [13] Endocrine, metabolic, or nutritional disease [13] Greater axis 1 comorbidity [16]
Clinical characteristics and trauma history	Impulsivity [1] Less suicidality [1] Higher baseline experiential avoidance, trait anxiety and anger [1] Length of illness and hospitalization history [1] Depression symptom severity in one study [1] No impact of baseline BPD, depression and overall symptom severity and general psychopathology [1]	Impulsivity [14] Anxiety [18] Bodily pain [13] Anger [16, 17] Non-acceptance of emotional responses [10] Experiential avoidance [17, 19] More than 86 weeks in a psychiatric hospital [15] Suicide attempts [15–17] (direction of effect unclear) Emotional abuse [20, 21] Less emotional neglect [20] Less physical neglect [21] No impact of childhood trauma [14]
Psychological and therapy process variables	Weaker therapeutic alliance [1] Commitment to change [1] Less internal and more external motivation for change [1] Higher perceived stigma [1] Less affective communication [1] Poor patient or therapist-rated therapeutic alliance [1] Longer stay duration [2] Study randomization [2] Outpatient setting [2] Availability of phone coaching [2]	Less frequent phone contacts [19] Availability of phone coaching [8] Weaker therapeutic alliance late in treatment [16] Change of therapist [21] DBT Consultation team [8]

In summary, treatment dropout is a relevant phenomenon in the treatment of BPD, and there is indication that the quality of the therapeutic alliance, especially in early treatment, is a predictive factor in DBT treatment. The purpose of this study was to replicate and expand findings of our previous study [21] by investigating, amongst other commonly examined variables, whether early patient-rated therapeutic alliance predicts dropout in a residential DBT treatment. We hypothesized that (1) a weak patient-rated therapeutic relationship is related to treatment dropout and that (2) a change of therapist between the preliminary talk and DBT treatment represents a rupture and therefore impairs the therapeutic alliance.

## Methods

### Recruitment

We approached all patients from age 18 to 65 that were discharged from our residential personality disorder unit between January 2019 and December 2021, and who fulfilled three or more criteria for BPD (Borderline Personality characteristics; BPC) as defined by DSM-5 (*n* = 44) to best represent a routine DBT sample. Exclusion criteria included the inability to consent, other severe mental disorders (bipolar disorder, acute psychosis), the inability or unwillingness to avoid alcohol, illicit or not prescribed drug use during the residential stay, the inability to negotiate a non-suicide agreement, ongoing traumatic contact with the perpetrator, and a Body Mass Index < 16.5. Most of these exclusion criteria are based on the ward’s requirements for treatment. Beyond that, we excluded patients with a treatment history on our ward which may have confounded the therapeutic alliance and the impact of the initial contact with a therapist in the DBT-briefing. Due to ethical guidelines, simultaneous participation in other treatment studies, as well as pregnancy or breastfeeding were also defined as exclusion criteria.

### Procedure

Within the first week of their residential stay, all participants who met the inclusion criteria were informed about the objectives and conditions of the study. They gave their written informed consent to participate in the study and to publish the results. The ethical standards were in line with the Declaration of Helsinki. Subsequently, each participant completed a set of questionnaires. Dropout status was documented at discharge (see definition of dropout).

### Measures

#### Clinical measures

Borderline symptom severity was assessed using the Borderline Symptom List [BSL; 30]. The BSL is a 95-item self-report measure for symptoms based on the DSM-IV criteria for BPD. The internal consistency of the BSL in our sample was α = 0.97.

The Beck Depression Inventory – Second Edition (BDI-II) was used to assess current depressive symptoms [31]. The BDI-II is a widely used 21-item self-report questionnaire with established reliability and validity (Cronbach’s Alpha in the current sample: α = 0.90).

The Childhood Trauma Questionnaire [CTQ; 32] is a retrospective self-report questionnaire of childhood maltreatment experiences. It covers emotional, physical, and sexual abuse as well as emotional and physical neglect. In our sample, internal consistency for the subscales of emotional abuse (α = 0.74), physical abuse (α = 0.79), sexual abuse (α = 0.74), and emotional neglect (α = 0.75) was adequate. However, Cronbach’s Alpha for physical neglect (α = 0.65) was marginal.

The Posttraumatic Diagnostic Scale [PDS; 33] was used to assess exposure to traumatic experiences via its checklist of 12 potentially traumatic events. The number of different traumatic events was used as a measure of trauma burden. The PDS is widely used and is known to be valid and reliable.

To assess the severity of general psychopathology, we administered the Symptom Checklist in the 90-items revised version [SCL-90-R; 34]. In the study at hand, we only report the Global Severity Index as a measure of the psychopathological burden (internal consistency: α = 0.97).

Dissociation was assessed using the Dissociative Experiences Scale [DES; 35], which is a 28-item self-report measure of the frequency of different dissociative experiences. Internal consistency was α = 0.91 in the current sample.

The World Health Organization Quality of Life Questionnaire in its brief version [WHOQOL-BREF; 36] was used to gain an overall impression of the quality of life and general health (internal consistency: α = 0.83).

#### Working alliance

The patient-rated therapeutic alliance was assessed via the short (12-item) version of the Working Alliance Inventory [WAI-SR; 24]. It consists of three subscales with four items each rated using a 5-point response scale: (1) agreement on the tasks of treatment, (2) agreement on the goals of treatment and (3) development of an affective bond. The WAI-SR is widely used and has been proven to show good psychometric properties in international as well as German inpatient and outpatient samples [37]. It is based on the pantheoretical, tripartite conceptualization of therapeutic alliance by Bordin [25]. Internal consistencies of the subscales task (α = 0.78), goal (α = 0.78), and bond (α = 0.76) were acceptable. Cronbach’s Alpha of the total score was α = 0.90.

## Treatment

The residential treatment in our personality disorder unit is certified by the German DBT Board of Certification (DDBT). As common in DBT settings, patients were seen in outpatient counseling sessions before starting DBT (DBT briefing). The briefing, which lasts one hour, includes examination of the patient, assessment of treatment history, indication for treatment, and assessment of both inclusion and exclusion criteria for treatment. As often as possible, the therapist who provided the briefing also provided the treatment, usually two to three months later. However, due to organizational reasons, this was not always possible; in these cases, another therapist took over after the DBT briefing (documented as a change of therapist). A change of therapist did not occur due to clinical considerations in any case. There were no further contacts after DBT briefing and DBT treatment. Patients were admitted to our ward approximately three months after the DBT briefing and were treated with DBT integrated in a standard residential setting for a total of eight to ten weeks. There was no change of therapist during treatment for any reason (organizational or clinical). Within the sixth week, the discharge date was determined depending on the patient’s progress, goals, and needs.

### Dialectical behavior therapy

DBT is a cognitive-behavioral treatment program that was developed to treat suicidal patients with BPD [6]. Over a period of eight to ten weeks, participants received weekly 50-minute individual treatment sessions (ten sessions over ten weeks) and weekly group therapies as follows: 180 min of skills training (24–30 sessions over ten weeks), 45 min of mindfulness training, and psychoeducation about BPD (8–10 sessions over ten weeks). The program is designed to help patients achieve the following therapeutic goals: (1) reduce suicidal behaviors, (2) reduce therapy-disrupting behaviors, and (3) reduce other risky or destabilizing behaviors. Standard DBT seeks to achieve these goals by (1) teaching behavioral skills, (2) motivating the application of these skills, (3) generalizing the learned skills to the patient’s natural environment, (4) structuring the treatment environment to reinforce functional behavior, and (5) teaching therapeutic resources and motivation to effectively treat patients with BPD.

### Therapeutic alliance in DBT

The therapeutic alliance in DBT is enforced by three dialectical principles. The patient-therapist relationship is seen as a “real” relationship, patient and therapist are equally affected by behavioral principles. Beyond that, DBT comprehends this relationship as a dynamic interaction that needs conflicts and conflict resolution as a process of change by constantly interweaving acceptance and change. The relationship in DBT is continuously both a tool to make the treatment work as well as itself having a therapeutic effect [38].

### Standard inpatient care

Standard Inpatient Care (SIC) included all non-specific therapeutic elements. Over an eight- to ten-week period, participants received 30-minute supportive conversations with the primary nurse twice a week, art or music therapy twice a week, and weekly body therapy. In addition, all patients received morning rounds, movement therapy and learned relaxation techniques. Patients also received standard psychopharmacological treatment, which was documented.

### Definition of dropout

To assess treatment discontinuation, we recorded whether the participant was discharged from our unit before week eight or before the final discharge date set at week six. The reasons for discontinuation were documented (by the patient or by the ward). In all cases, contingency management was the reason for discharge on part of the ward. It includes positive consequences for functional behaviors to reinforce these and increase the likelihood of their occurrence, as well as negative consequences for dysfunctional behaviors to extinguish these. Although dysfunctional behavior patterns are part of the clinical picture of BPD, reducing them is necessary in order to reduce negative consequences for the patients themselves and others on the ward, and furthermore to enable the use of adaptive strategies in the first place. Dysfunctional behaviors, on the one hand, depend on the goals of the patients. On the other hand, some dysfunctional behaviors are defined according to the DBT hierarchy: suicidal behaviors, severe non-suicidal self-injuries, drug use, and therapy-disrupting behaviors (missing sessions, violating general ward rules). When dysfunctional behavior occurs repeatedly (usually four times), a patient is discharged from treatment. If maladaptive behavior occurs, intensive work is done to build up adaptive problem-solving strategies. If maladaptive strategies repeatedly occur despite intensive coaching, treatment is discontinued (assuming that the therapy in its current form or at the current time is not helpful). The more a behavior is harmful to other patients on the ward, the sooner a patient will be discharged (e.g., assaulting a fellow patient leads to immediate discharge).

### Data analyses

The initial analyses included group comparisons with Mann-Whitney-U-tests and χ² statistics as well as exploratory correlation analyses. These statistics were obtained using SPSS 25 [39]. Variables of interest included in further analyses were, first, available variables that showed significant associations in the literature and our previous study [21] or that appeared relevant through the exploratory analyses. All variables of interest were mean-centered or dummy-coded depending on the level of scale. We used a multilevel logistic regression (patients nested within therapists) to predict dropout from DBT treatment. The data were analyzed with R Studio [40] and the package “lme4” [41]. A random intercept was chosen to allow therapists to differ in their dropout rates. We used a consecutive variable selection approach based on five blocks of variables (socio-demographics, comorbid disorders, clinical characteristics, trauma history and treatment alliance) to determine the additional proportion of variance explained by this set of variables. Based on these block-wise analyses, only predictors showing a p < .10 were included in the final model to increase power. In the final model, only predictors with a p < .05 were interpreted as statistically significant. To avoid multicollinearity, only the total score of the WAI was included in the analyses. Because the data were collected in a naturalistic setting, there were some missing values in the predictor variables examined. Therefore, we ran the final model with both list-wise deletion and multiple imputations and compared the results. Missing data were imputed using the package “mice” [42] based on five different imputed datasets.

## Results

### Sample characteristics and dropout

The sample consisted of 44 treatment-seeking patients with borderline pathology. Participants had an average age of 28.2 years (*SD* = 8.60), 79.5% were female (*n* = 35). 28.5% of the participants were currently living in a relationship and reported an average of 10.8 years of basic school education (*SD* = 1.60). 86.4% (*n* = 38) fulfilled the diagnostic criteria of BPD, 13.6% (*n* = 6) showed borderline characteristics only (BPC; 3 or 4 BPD-criteria).

The dropout rate was 34.1% (*n* = 15) and the mean duration of treatment was 35.13 days (*SD* = 17.81) for the dropout group and 62.31 days (*SD* = 12.01) for the completer group. Of the patients who discontinued treatment, 53.3% were discharged by the ward and 46.7% discontinued at their own request (see supplementary material). 75.0% (*n* = 33) of patients experienced a change of therapist between DBT-briefing and treatment. Comparisons of the dropout vs. completer group showed that both groups did not differ with regard to demographic characteristics and pretreatment symptom severity except for the WAI-SR total score and by trend, all subscales of the WAI-SR (with higher levels in the completer group; see Table 2). Reasons for dropout are presented in Table 3.

**Table 2:** Group comparison of treatment completer and dropout group

	Group				Statistic	
Characteristic	Completers (*n* = 29)		Non-Completers (*n* = 15)			
	*Mean*	*SD*	*Mean*	*SD*	*Z*	*p*
Age (years)	28.07	7.61	28.47	10.55	− 0.521	0.602
School (years)	10.93	1.62	10.40	1.55	− 0.990	0.322
Number of comorbidities (Axis 1 and 2)	1.41	0.86	1.47	1.25	− 0.129	0.897
	* N*	*%*	*N*	*%*	*χ²*	*p*
Sex (female)	23	82.10	12	80.00	0.003	0.957
Current BPD	24	82.80	14	93.30	0.939	0.333
Current PTSD	10	34.50	4	26.70	0.278	0.598
Current substance use disorder	3	10.30	4	26.70	1.969	0.161
	*Mean*	*SD*	*Mean*	*SD*	*Z*	*p*
Global Severity Index (SCL-90-R)	1.54	0.71	1.62	0.53	− 0.433	0.664
BSL Total Score	1.90	0.78	2.15	0.56	− 1.028	0.304
BDI-II Total Score	31.76	12.58	34.53	8.72	− 0.372	0.710
DES Mean Score	22.97	14.72	22.50	12.74	− 0.037	0.970
Number of traumatic event types (PDS)	2.83	1.54	3.36	1.91	− 0.848	0.397
Childhood Maltreatment (CTQ)						
Emotional Abuse (Sum Score)	16.69	4.75	16.93	5.47	− 0.285	0.776
Physical Abuse (Sum Score)	8.62	4.77	8.00	4.22	− 0.160	0.873
Sexual Abuse (Sum Score)	7.73	3.60	7.50	2.85	− 0.119	0.905
Emotional Neglect (Sum Score)	16.42	4.47	14.57	5.19	− 0.853	0.394
Physical Neglect (Sum Score)	9.46	4.09	9.93	2.84	− 1.015	0.310
Treatment Process Variables						
Therapeutic Alliance (WAI-SR Total Score)	45.52	8.16	35.60	10.49	− 2.850	0.004*
WAI-SR – Task (Sum Score)	15.10	2.90	12.93	2.79	− 2.346	0.019
WAI-SR – Bond (Sum Score)	14.48	2.90	10.53	5.21	− 2.473	0.013
WAI-SR – Goal (Sum Score)	15.93	3.17	12.13	3.94	− 3.099	0.002*
	*N*	*%*	*N*	*%*	*χ²*	*p*
Change of therapist	20	69.00	13	86.70	1.652	0.199

**Note.** BPD = Borderline Personality Disorder; PTSD = Posttraumatic Stress Disorder; SCL = Symptom Checklist, BSL = Borderline Symptom List, BDI = Beck Depression Inventory, DES = Dissociative Experiences Scale, PDS = Posttraumatic Diagnostic Scale, CTQ = Childhood Trauma Questionnaire, WAI-SR = Working Alliance Inventory-Short Revised. * *p* ≤ .010 (*p*-value Bonferroni-corrected for comparisons of treatment process variables)

**Table 3:** Reasons for DBT dropout (case by case)

	Discharge on part of the ward (*n* = 8)	Discharge on patient’s request (*n* = 7)
Reasons for dropout	1. Suicidality	1. New job offer
	2. Suicidality	2. Struggled with COVID-19-restrictons
	3. Aggressive behavior	3. Struggled with patient group
	4. Dissocial behavior in the patient group	4. Preferred outpatient treatment
	5. Repeated conflicts in the patient group	5. Felt wronged by team members and wanted to consume drugs again
	6. Rejection of treatment strategies and avoidance of appointments	6. Struggled with COVID-19-restrictons
	7. Repeated therapy-disrupting behavior in the skills group	7. Sense of having learned sufficient skills
	8. Suicidality	

### Predictors of dropout

Exploratory correlation analyses between treatment dropout and applied measures revealed a significant negative association only between WAI-SR sum score and dropout (*r* = − .471, *p* < .001) as well as all WAI-SR subscales and dropout (*r* ≥ − .345, *p* ≤ .022). All other measures (e.g., subscales of the Borderline Symptom List) did not correlate with treatment dropout. A change of therapist was not significantly correlated with the WAI-SR sum score (*r* = − .208, *p* = .176) or the subscales “task” and “bond” (*r* = − .088 − .195, *p* = .087 − .569). There was a trend towards a correlation between change of therapist and the WAI-SR subscale “goal” (*r* = − .261, *p* = .087).

Five logistic regressions with different predictor ranges were conducted below to predict dropout, resulting in one final model.

*Impact of demographic variables.* The model examining socio-demographics included sex and years of education. None of the variables were significant predictors of dropout (Table 4).

**Table 4:** Fixed Effects on dropout with demographic variables

	Estimate	Std. Error	*z*	*p*
Intercept	− 1.552	1.768	− 0.878	0.380
Sex	0.525	0.942	0.557	0.577
Years of education	− 0.257	0.399	− 0.644	0.519

**Note.** No significant results

*Impact of (comorbid) diagnoses.* The model examining (comorbid) diagnoses included the presence of full-blown borderline disorder (vs. -characteristics), the presence of PTSD (vs. no PTSD), the presence of substance use disorder (vs. no substance use disorder), and the number of comorbid conditions (including comorbid personality disorders in addition to borderline disorder/-characteristics). None of the variables had significant predictive value (Table 5).

**Table 5:** Fixed Effects on dropout with (comorbid) disorders

	Estimate	Std. Error	*z*	*p*
Intercept	− 0.300	1.704	− 0.176	0.860
BPD	− 0.391	1.340	− 0.292	0.770
PTSD	− 0.461	0.854	− 0.539	0.590
Substance Use Disorder	1.622	1.115	1.455	0.146
Number of Comorbidities	0.039	0.417	0.093	0.926

**Note.** BPD = Borderline Personality Disorder, PTSD = Posttraumatic Stress Disorder; no significant results

*Impact of childhood trauma severity.* The model examining childhood trauma severity included all subscales of the childhood trauma questionnaire, emotional and physical neglect and abuse as well as sexual abuse. Emotional and physical neglect predicted dropout significantly (Table 6). Higher values on physical neglect were associated with an increased risk for dropout, whereas higher values on emotional abuse were a protective factor against dropout.

**Table 6:** Fixed Effects on dropout with childhood trauma severity

	Estimate	Std. Error	*z*	*p*
Intercept	− 0.618	0.527	− 1.175	0.240
CTQ Emotional Abuse Sum Score	0.264	0.490	0.540	0.589
CTQ Physical Abuse Sum Score	− 0.337	0.495	− 0.681	0.496
CTQ Sexual Abuse Sum Score	0.164	0.468	0.351	0.726
CTQ Emotional Neglect Sum Score	− 1.225	0.662	− 1.849	0.064*
CTQ Physical Neglect Sum Score	1.110	0.656	1.691	0.091*

**Note.** CTQ = Childhood Trauma Questionnaire, *p < .10

*Impact of clinical characteristics and quality of life.* The model examining clinical characteristics and quality of life included all clinical measures (BDI-II, BSL, SCL, DES and WHOQOL). None of the variables had significant predictive value (Table 7).

**Table 7:** Fixed Effects on dropout with clinical characteristics and quality of life

	Estimate	Std. Error	z	p
Intercept	− 0.737	0.496	− 1.486	0.137
BDI-II Total Score	0.318	0.602	0.529	0.597
BSL Total Score	0.636	0.699	0.910	0.363
General Severity Index (SCL)	− 0.457	0.702	− 0.651	0.515
DES Total Score	− 0.470	0.492	− 0.956	0.339
WHOQOL Total Score	− 0.141	0.430	− 0.327	0.744

**Note.** BDI-II = Beck Depression Inventory II, BSL = Borderline Symptom List, SCL = Symptom Checklist, DES = Dissociative Experience Scale, WHOQOL = World Health Organization Quality of Life Questionnaire; no significant results

*Impact of therapeutic alliance.* The model examining therapeutic alliance included the mean score of the WAI-SR as well as a variable coding a change of therapist. Only the WAI significantly predicted dropout (Table 8). Higher values were accompanied by a decreased risk of dropout.

**Table 8:** Fixed Effects on dropout with therapeutic alliance

	Estimate	Std. Error	*z*	*p*
Intercept	− 0.500	0.593	− 0.842	0.400
WAI-SR Total Score	− 1.119	0.472	− 2.371	0.018*
Change of Therapist	− 0.986	1.144	− 0.862	0.389

**Note.** WAI-SR = Working Alliance Inventory-Short Revised, *p < .05

*Final model predicting dropout.* In the final prediction model including all important (*p* < .10) predictors from the previous models, only the WAI-SR significantly predicted dropout (Table 9). The results remained mainly the same when estimating the final model using multiple imputation of missing values instead of list-wise deletion (Table 10).

**Table 9:** Fixed Effects on dropout (final model)

	Estimate	Std. Error	*z*	*p*
Intercept	− 0.863	0.490	− 1.760	0.079
CTQ Physical Neglect Sum Score	1.022	0.652	1.569	0.117
CTQ Emotional Neglect Sum Score	− 1.194	0.641	− 1.862	0.063
WAI-SR Total Score	− 1.111	0.493	− 2.254	0.024*

**Note.** CTQ = Childhood Trauma Questionnaire, WAI-SR = Working Alliance Inventory-Short Revised, *p < .05

**Table 10:** Fixed Effects on dropout (final model) with multiple imputed values

	Estimate	Std. Error	*z*	*p*
Intercept	− 0.806	0.533	− 1.514	0.130
CTQ Physical Neglect Total Score	0.851	0.599	1.421	0.155
CTQ Emotional Neglect Total Score	− 0.863	0.551	− 1.565	0.118
WAI-SR Total Score	− 1.006	0.452	− 2.225	0.026*

**Note.** CTQ = Childhood Trauma Questionnaire, WAI-SR = Working Alliance Inventory–Short Revised, *p < .05

## Discussion

The purpose of this study was to investigate whether early patient-rated therapeutic alliance is related to dropout in a residential DBT treatment. It is a follow-up to a previous study which showed that a change of therapist between DBT-briefing and -treatment increased the dropout rate of DBT [21]. As this result was interpreted as an impairment of the therapeutic alliance, the present study aimed to assess therapeutic alliance and to investigate its effect on dropout from 10-week inpatient DBT among patients with borderline pathology. Our hypotheses were partially confirmed. Therapeutic alliance early in treatment predicted later DBT dropout. However, therapeutic alliance was not significantly associated with a change of therapist (*r* = .17).

Our results support the importance of therapy process variables, and therapeutic alliance in particular, as predictors of therapy dropout in borderline pathology. Patients who demonstrated a stronger therapeutic relationship at baseline had a lower risk of dropout over the course of treatment. This finding extends the existing literature finding that a poor therapeutic alliance predicts dropout in DBT [16]. Our results, like those of Spinhoven et al. [29], suggest that the therapeutic relationship early in therapy is particularly important for the course of therapy.

In order to [43, 44] improve the therapeutic alliance early in treatment, it would be interesting to disentangle trait- and state-like components of the therapeutic alliance [45]. In our study, it is unclear whether the therapeutic alliance captured during the first week of treatment is already affected by DBT strategies (state) or depends on the patients’ basic bonding abilities (trait) or both. A more precise assessment of state- and trait-like components and knowledge on how to respond to both components could provide clues on how to improve the therapeutic alliance for each patient individually in the future.

DBT claims to have a specific influence on the therapeutic alliance, that is, to particularly enhance the state-like alliance. In a study by Bedics et al. [46], DBT therapists reported more working strategy consensus early in treatment and an overall higher alliance during treatment as compared to non-behavioral psychotherapy for BPD. Overall, there were no significant differences between treatment conditions in patient ratings of the alliance. Nevertheless, most results suggested that elements of the patient-rated alliance work differently in DBT and have unique effects on particular outcomes. For example, an increase in the patient-rated alliance was associated with reduced non-suicidal self-injuries in DBT but not in non-behavioral psychotherapy for BPD.

Furthermore, regular monitoring of the therapeutic alliance may be useful to improve treatment. In a multilevel meta-analysis, feedback on patients’ progress (including the therapeutic alliance in some of the studies reviewed) to the therapists has been found to improve treatment and decrease dropout rates [47]. However, feedback may not be as effective in patients with personality disorders or even cause adverse effects in Cluster B personality disorders [48]. McMain et al. [49] name five strategies to grow a positive therapeutic alliance: (a) cultivating emotional awareness, (b) structuring treatment, (c) being responsive, (d) supervision or team involvement, and (e) exploring ruptures. Further research is needed to identify influencing factors to improve the therapeutic alliance during treatment.

Different from what we expected, the strength of the therapeutic relationship was not significantly related to a change of therapist in our study. This result could mean that the change of therapist puts less strain on the therapeutic relationship than primarily assumed. However, the proportion of patients for whom change of therapist was necessary due to organizational reasons on our ward was higher in the present study (75%) than in our previous study [21]. The change of therapist between preliminary conversation and treatment could thus be understood as the rule rather than the exception in the patients’ experience (and subsequently rejection) compared to other patients on the ward. Yet, the sample is smaller than in the preliminary study, thus a small effect may still be present but not visible due to insufficient power.

Comparable to previous studies, dropout rate was not associated with socio-demographic variables [1]. Also, traumatic experiences showed no significant predictive value. However, the small sample size must also be noted here. The dropout rate found in our study (34.1%) was higher than in a recent DBT meta-analysis [28%; 8] and our own previous study [24.7%; 21], which may be an impact of the COVID pandemic due to exit restrictions in our clinic.

A strength of this study is that we captured reasons for treatment discontinuation. Dropout on part of the patient and on part of the therapeutic team is relatively equally distributed. This result is comparable to our previous study [21]. Qualitative methods would be helpful to understand the process of treatment discontinuation in detail and derive accurate clinical implications.

The study has several limitations, some of which are associated with the naturalistic setting of the study. First, only clinical diagnoses were made; no detailed diagnostics were performed using diagnostic interviews and independent raters. Therefore, previously known predictors such as PTSD, substance abuse and the number of BPD symptoms may not have been picked up. The sample is naturally very heterogeneous. We only used self-report measures, also for capturing the therapeutic relationship. This makes missing values more likely.

Moreover, the therapeutic relationship was recorded one-sidedly by the patients. However, in psychotherapy research, the patient’s (not the therapist’s) judgment of the therapeutic alliance is particularly important. Not all patients chose to participate in the study and complete the questionnaires. Patients who chose not to participate may have special characteristics that affect the generalizability of the results. Furthermore, the sample size is very small; the results require replication. In addition, an impact of the COVID pandemic on dropout rates and reasons for dropout cannot be ruled out.

Future research should replicate and further examine the effects of the therapeutic relationship rated by both patients and therapists on dropout and also treatment outcome in a larger sample. In addition, the improvement of the therapeutic relationship in general or certain aspects of it (e.g., subscale of the WAI) through feedback of the patient-rated therapeutic relationship to the therapists in DBT should be examined. Beyond that, other treatment process variables, such as expectations of treatment, should be included.

## Conclusions

This study highlights the importance of the therapeutic relationship within the first week of treatment for therapy dropout rates in DBT. Learning about the causes and influencing factors of treatment discontinuation in BPD is important to improve treatment retention. Here, the therapeutic alliance appears to be one important component. Monitoring and strengthening the patient-therapist alliance may be an important mechanism of change for patients and could make DBT accessible to as many patients as possible. This would allow more patients to benefit from this highly effective treatment.

## Data Availability

The dataset supporting the conclusions of this article is shared upon request directed to Carolin Steuwe (carolin.steuwe@evkb.de).

## Abbreviations

BDI-II: Beck Depression Inventory 2nd Edition
BPC: Borderline Personality Characteristics
BPD: Borderline Personality Disorder
CTQ: Childhood Trauma Questionnaire
DBT: Dialectical Behavior Therapy
DDBT: DBT Board of Certification
DES: Dissociative Experiences Scale
DSM: Diagnostic and Statistical Manual of Mental Disorders
GSI: Global Severity Index (SCL-90-R)
PDS: Posttraumatic Stress Diagnostic Scale
PTSD: Posttraumatic Stress Disorder
SCL-90-R: Symptom-Checklist 90 items, revised version
SIC: Standard Inpatient Care
WAI-SR: Working Alliance Inventory-Short Revised
WHOQOL: World Health Quality of Life Questionnaire
